# Pain control according to the periprostatic nerve block site in magnetic resonance imaging/transrectal targeted prostate biopsy

**DOI:** 10.1038/s41598-022-04795-x

**Published:** 2022-01-14

**Authors:** Jeong Woo Yoo, Kyo Chul Koo, Byung Ha Chung, Kwang Suk Lee

**Affiliations:** Department of Urology, Gangnam Severance Hospital, Yonsei University College of Medicine, 211 Eonju-ro, Gangnam-gu, Seoul, 06273 Republic of Korea

**Keywords:** Medical research, Oncology, Urology

## Abstract

We analyzed the intensity of pain at each site of systemic prostate biopsy (SBx) and compared the intensity of pain among magnetic resonance (MRI)-targeted transrectal biopsies according to the periprostatic nerve block (PNB) site. We collected data from 229 consecutive patients who had undergone MRI-targeted biopsy. Patients were stratified into two groups according to the site of PNB (base versus base and apex PNB). Pain was quantified at the following time points: probe insertion, injection at the prostate base, injection at the prostate apex, MRI cognitive biopsy (CBx), MRI/transrectal ultrasound fusion biopsy (FBx), SBx, and 15 min after biopsy. For all biopsy methods, the average pain were significantly higher in the base PNB group than in the base and apex PNB group (CBx, *p* < 0.001; FBx, *p* = 0.015; SBx, *p* < 0.001). In the base and apex PNB group, FBx was significantly more painful than SBx (*p* = 0.024). Overall, regardless of the PNB site, pain at the anterior sites was more than that at the posterior sites in FBx (*p* = 0.039). Base and apex PNB provided better overall pain control than base-only PNB in all biopsy methods. In the base and apex PNB group, FBx was more painful than CBx and SBx.

## Introduction

Prebiopsy prostate magnetic resonance imaging (MRI) is recommended by the National Comprehensive Cancer Network (NCCN) to determine the need for performing prostate biopsy. Prostate MRI is more sensitive in detecting clinically significant prostate cancer than systemic prostate biopsy (SBx). Prebiopsy MRI is helpful in identifying lesions in not only the anterior prostate but also in the apex of the prostate; these lesions are not routinely assessed using SBx because of the associated challenges^1^. With the increasing applications of prebiopsy MRI, the MRI/transrectal ultrasound (TRUS) fusion targeted prostate biopsy (FBx) has been recommended by the NCCN owing to its improved accuracy^2^.

NCCN also recommends the use of local anesthesia to reduce pain during prostate biopsy^2^. A combination of intrarectal lidocaine gel and periprostatic nerve block (PNB) is useful during SBx^3–8^. Base PNB is the most common method of PNB. Major neurovascular bundles of the prostate pass through the base PNB site and a large portion of the prostate is anesthetized by base PNB^9^. The apex PNB blocks the somatic nerve in the apex of the prostate, one of the most painful areas during biopsy^9^. The apex PNB is usually performed before local transperineal biopsy^10,11^. As the location of the target lesion in FBx is different from that in SBx, pain intensity in SBx should be predicted according to the biopsy site, and the optimal local anaesthesia method for FBx should be used. However, to our best knowledge, no previous study has analyzed the differences in pain intensity according to the biopsy site, and only a few have assessed the optimal local anaesthesia method for FBx.

Therefore, we investigated pain intensity during various biopsies according to the biopsy site and compared the pain alleviation during various biopsy methods, including MRI cognitive targeted prostate biopsy (CBx), FBx, and SBx, according to the site of PNB.

## Results

### Baseline characteristics

Patient characteristics according to the PNB site are presented in Table 1. There were no differences in age, prostate-specific antigen (PSA) level, prostate volume, or history of prostate biopsy between the base PNB and base and apex PNB groups.

**Table 1:** Baseline characteristics of groups according to the site of the PNB.

	Base	Base and apex	*p*
N	110 (49.8)	111 (50.2)	
Age (years)	69.0 ± 8.2	68.86 ± 7.8	0.839
PSA (ng/mL)	8.14 (5.42–13.63)	6.92 (4.78–10.23)	0.181
Prostate volume (mL)	35.7 ± 16.1	37.0 ± 18.1	0.568
History of prostate biopsy	20 (18.2)	12 (10.8)	0.120
**PI-RADS score on target**	132	150	< 0.001
3	40 (30.3)	34 (22.7)	
4	47 (35.6)	88 (58.7)	
5	45 (34.1)	28 (18.7)	
Prostate cancer detection rate	85 (77.3)	79 (71.2)	0.299
Prostate cancer detection on target	90 (68.2)	89 (59.3)	0.124
**ISUP grade group**			0.132
1	15 (13.6)	19 (17.1)	
≥ 2	70 (63.6)	60 (54.1)	
**Time (s)**			
Periprostatic nerve block	149.5 ± 47.2	160.0 ± 53.3	0.121
Prostate biopsies	285.8 ± 165.2	327.6 ± 367.5	0.277
**Adverse events**			
Vasovagal syncope	1 (0.9)	0 (0.0)	0.316
Allergic reaction	0 (0.0)	0 (0.0)	–
AUR	0 (0.0)	0 (0.0)	–
Urinary retention because of blood clot	0 (0.0)	0 (0.0)	–
Fever	0 (0.0)	0 (0.0)	–

Data are expressed as number (%), mean ± standard deviation, and median (IQR range).

*AUR* acute urinary retention, *ISUP* International Society of Urological Pathology, *IQR* interquartile, *PI-RADS* prostate imaging-reporting and data system, *PNB* periprostatic nerve block, *PSA* prostate-specific antigen.

### Visual analog scale scores during the biopsy

**Figure 1:**
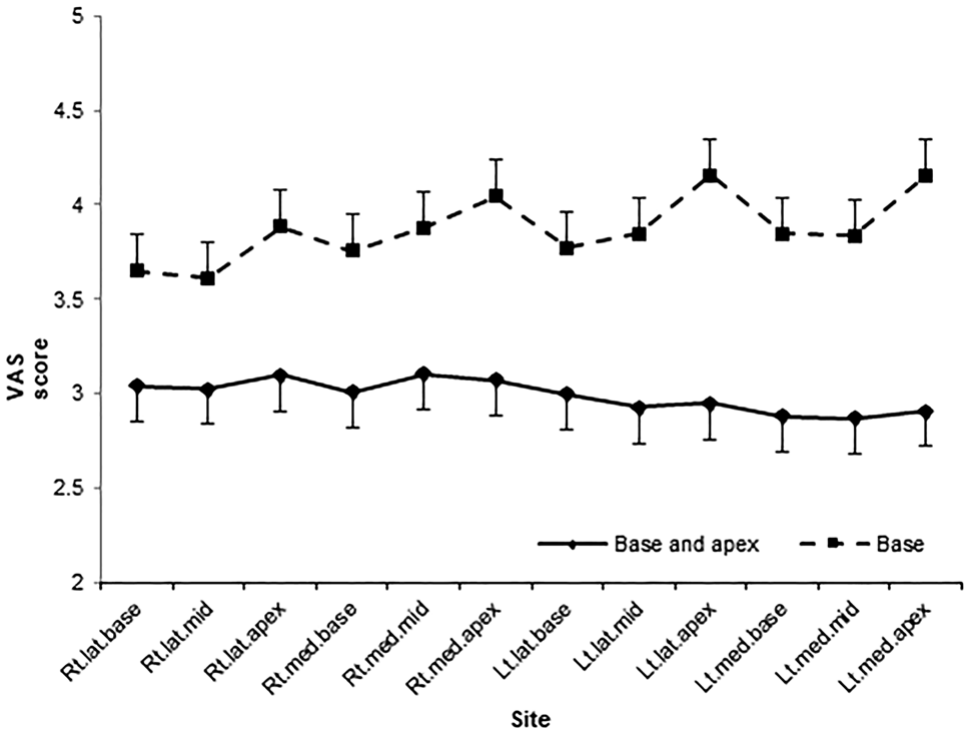
Mean profile plot of VAS scores for each SBx. *SBx* systemic prostate biopsy, *Lat* lateral, *Lt* left, *Med* medial, *Rt* right, *VAS* visual analog scale.

The visual analog scale (VAS) scores according to the PNB site are presented in Table 2. There were no significant differences in VAS scores at different time points (probe insertion, injection at the prostate base, injection at the prostate apex, and 15 min after prostate biopsy) between the base PNB and base and apex PNB groups. The differences in VAS scores for each core biopsy of SBx between the base PNB and base and apex PNB groups are presented in Fig. 1. In the base PNB group, pain at the apex lesions was more than that at the order lesions (*p* < 0.001, Fig. 1). For all biopsy methods, patients in the base and apex PNB group reported lower VAS scores than those in the base PNB group (Table 2).

**Table 2:** Mean VAS scores during prostate biopsy in patients according to the site of the PNB.

VAS scores	Base	Base and apex	*p*
Probe insertion	3.91 ± 2.03	3.45 ± 2.47	0.501
Injection at base^a^	2.80 ± 1.86	2.92 ± 1.91	0.652
Injection at apex^b^	4.30 ± 2.33	4.75 ± 2.53	0.177
CBx^c^	4.06 ± 2.59	3.01 ± 2.26	< 0.001
FBx^d^	3.91 ± 2.44	3.21 ± 2.39	0.015
SBx^e^	4.00 ± 2.66	2.88 ± 2.19	< 0.001
At 15 min post prostate biopsy	0.15 ± 0.62	0.20 ± 0.85	0.598

Data are expressed as mean ± SD.

^a^Mean and SD of VAS scores during prostate base injections.

^b^Mean and SD of VAS scores during prostate apex injections.

^c^Mean and SD of VAS scores for individual CBx.

^d^Mean and SD of VAS scores for individual FBx.

^e^Mean and SD of VAS scores for individual SBx.

*CBx* magnetic resonance imaging cognitive target prostate biopsy, *FBx* magnetic resonance imaging/transrectal ultrasound fusion targeted prostate biopsy, *SBx* prostate systemic biopsy, *PNB* periprostatic nerve block, *SD* standard deviation, *VAS* visual analog scale.

In the base and apex PNB group, FBx was painful than CBx and SBx. FBx was significantly more painful than SBx (3.21 vs. 2.88, *p* = 0.024) and marginally more painful than CBx (*p* = 0.104). Between CBx and SBx, there was no significant difference in VAS scores (*p* = 0.327). In the base PNB group, there was no difference in VAS scores among the biopsy methods.

We compared the pain intensity between the anterior and posterior sites (Table 3). In FBx, anterior site biopsy was significantly more painful than posterior site biopsy in all patients regardless of the PNB site (*p* = 0.039). In the base PNB group, no differences in pain were found between the anterior and posterior sites (*p* = 0.069). Patients in the base and apex PNB group reported lesser pain in the anterior site biopsy than those in the base PNB group, and the difference was greater for FBx than for CBx.

**Table 3:** Differences in mean VAS scores during various types of biopsies between the two groups according to biopsy sites.

VAS pain scores	Anterior	Posterior	*p*
CBx with base PNB	4.58 ± 2.58	3.98 ± 2.59	0.292
CBx with base and apex PNB	3.59 ± 2.55	2.89 ± 2.14	0.155
Overall CBx	4.02 ± 2.59	3.42 ± 2.43	0.098
FBx with base PNB	4.87 ± 2.93	3.71 ± 2.26	0.069
FBx with base and apex PNB	3.72 ± 2.67	3.09 ± 2.30	0.177
Overall FBx	4.22 ± 2.82	3.39 ± 2.29	0.039

Data are expressed as mean ± SD.

*CBx* magnetic resonance imaging cognitive target prostate biopsy, *FBx* magnetic resonance imaging/transrectal ultrasound fusion targeted prostate biopsy, *PNB* periprostatic nerve block, *SD* standard deviation, *VAS* visual analog scale.

### Complications

One patient (0.9%) showed vasovagal syncope in the base PNB group, but recovered without any medical therapy. No major complications were observed in the base and apex PNB group (Table 1).

## Discussion

Here, we confirmed the efficacy of additional PNB performed at the prostate apex during CBx and FBx, based on previous studies on local anaesthesia in SBx, by comparing the differences in pain intensity among various biopsy methods. FBx was significantly more painful than other biopsy methods in the base and apex PNB group. We compared the difference in the pain intensity during anterior and posterior site biopsies and observed significant differences during FBx and marginally significant differences during CBx. Our findings can help in the selection of the optimal local anaesthesia method according to the core biopsy site, biopsy method, and number of core biopsies.

Studies have compared the pain intensity between FBx and SBx and reported that SBx is relatively more painful than FBx, which contradicts our results^12–14^. Demirtas et al. reported VAS scores during FBx and SBx as 2.0 (1.0–4.0) and 3.0 (1.0–5.0), respectively, but the scores were recorded 5 min after the procedure^12^. Egbers et al. reported VAS scores during FBx and SBx as 2.0 (0.0–7.0) and 3.0 (0.0–9.0), respectively, but the scores were recorded 1 week after the procedure via a telephone^13^. Kasivisvanathan et al. reported VAS scores of 1.0 (0.0–3.0) for FBx and 2.0 (1.0–4.0) for SBx, but they included patients in whom only MRI was performed without biopsy in the FBx group^14^. Because of these differences, VAS scores during FBx and SBx were underestimated in these previous studies compared to our study. In a previous literature review, VAS scores during SBx in the base PNB group ranged from 3.37 to 4.97^15–17^. In our study, the mean VAS score (4.00) during SBx was similar to that in the aforementioned studies.

We found that biopsy in the anterior site was more painful than that in the posterior site. The base PNB blocks the nerve originating from the presacral and hypogastric plexuses, and the apex PNB blocks the somatic nerve originating from the pudendal canal, but not all nerves. There are a few other nerve fibers on the anterior and superolateral of prostate^9^. Moreover, prostate size is associated with pain during SBx, and the pain intensity is greater during SBx of an enlarged prostate due to the longer distance between the local anesthesia and biopsy sites^4,18^. The distance between the PNB and biopsy sites affects adequate pain control. Additional apex PNB leading to a lower intensity of overall pain is associated with the decreased distance between the PNB and biopsy sites. As the site for PNB is located posteriorly, the anterior site was associated with a greater pain intensity.

VAS scores during anterior site biopsy were lower in the base and apex PNB group than in the base PNB group. The additional apex PNB could have reduced the intensity of anterior site pain. Apex PNB anesthetizes the somatic branch of the inferior rectal nerve from the pudendal nerve and hence reduces the pain intensity below the dentate line, which is the site of needle puncture during apex or anterior prostate biopsy^19^. Although the difference in the pain intensity between the two groups was not significant in our study, more meaningful results could be obtained through subsequent studies with larger sample sizes.

Core biopsies in FBx or CBx are mostly performed in sites that are relatively far from the rectum, such as the anterior prostate, or sites that are at a greater angulation with the natural orientation of the rectum, such as the prostate margin, which are not performed routinely in SBx^20^. Therefore, the probe could impinge on the rectum, leading to greater pain during FBx. FBx was marginally more painful than CBx because the manipulation speed of the freehand is greater and the manipulation is smoother in CBx than in the fusion system of FBx. This is in line with the result of a previous study that probe manipulation could induce pain during biopsy^12,21^. Considering that the prostate volume in Caucasian men is larger than that in Asian men, the angle of manipulation of the probe for biopsy of the apex or anterior site is larger, and the distance from the PNB site to the apex or anterior prostate is greater. Therefore, the pain may be more severe in Caucasian men^22^.

Our results showed no significant difference in the pain intensity according to the biopsy methods in the base PNB group, but pain during FBx in the base and apex PNB group was the most severe. The overall pain was more with all methods in the base PNB group, but the base and apex PNB group exhibited relatively good overall pain control. Therefore, patients in this group were more sensitive to pain during probe manipulation. Considering that the pain tended to be more severe as the biopsy proceeded, FBx is considered more painful than SBx^9,23^.

The present study was planned to determine the efficacy of additional apex PNB in the recent era of MRI-targeted biopsy. We found meaningful results on the difference in pain intensity according to the biopsy method with PNB, which have not been demonstrated previously. Nonetheless, our study is not without limitations. First, this is a single-center retrospective pilot study with a relatively small sample size. The patients were blinded as to the methods of nerve block given, but physician was not blinded, therefore the results are not free of bias. In addition, consensus on pain intensity and the possibility of complications due to additional apex injections is needed. Moreover, even if the average pain of each puncture is lower, it is difficult to directly compare the pain level of each method with different core counts. We performed three biopsy methods sequentially, and there was a limitation in directly comparing pain between each biopsy method. Finally, as we attempted to analyze the difference in pain intensity by matching the target site corresponding to the SBx site, obtaining meaningful results was difficult owing to the small sample size. We plan to validate our results through a well-controlled, prospective, double-blind, randomized, multi-center study.

Prostate biopsy methods have undergone innovative changes, such as performing CBx or FBx with SBx. To increase the cancer diagnosis rate, FBx or CBx is often performed with SBx. Therefore, the optimal method of local anaesthesia should be determined based on the procedure planned and the site of the procedure. Additional PNB administered in the prostate apex provides better overall pain control in CBx, FBx, and SBx.

## Methods

### Ethic approval

This study was approved by the institutional ethics committee (Yonsei university health system, Seoul, Korea, 3-2019-0418), and all procedures were conducted in accordance with the ethical standards of the 1964 Declaration of Helsinki and its later amendments. The informed consent requirement was waived by ethics committee of Yonsei university health system because this study was based on retrospective, anonymous patient data and did not involve patient intervention or the use of human tissue samples.

### Patient selection

**Figure 2:**
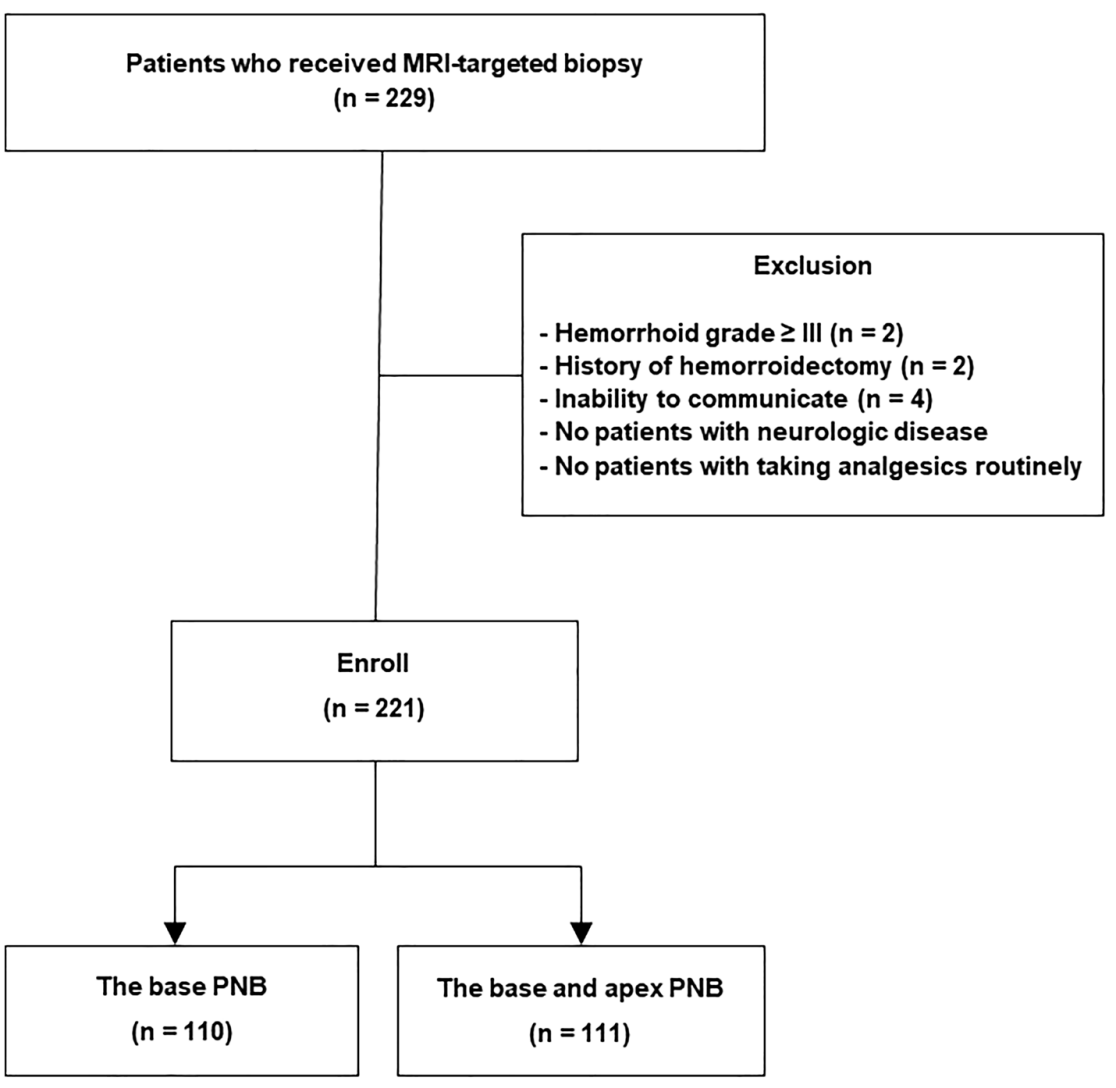
Study cohort flow diagram. *MRI* magnetic resonance image, *PNB* periprostatic nerve block, *TRUS* transrectal ultrasound.

We prospectively collected data from 229 consecutive patients who underwent MRI-targeted transrectal biopsy between January 2019 and September 2020. Patients who were unable to receive transrectal ultrasound probe injection, had severe haemorrhoids (Grade ≥ III, n = 2), had undergone related surgery (n = 2), and were unable to communicate (n = 4) were excluded. According to prebiopsy history taking, there were no patients with neurologic disease such as paraplegia or hemiplegia, and no patients with chronic pain who took analgesics routinely. Finally, 221 (96.5%) patients were selected for analysis (Fig. 2).

### Data collection

Patient data pertaining to age, PSA level, prostate volume, history of prostate biopsy, prostate imaging-reporting and data system (PI-RADS) scores, pathology results, time required for PNB and biopsies, adverse events (vasovagal syncope, allergic reaction, acute urine retention, urinary retention because of blood clot, and fever), VAS (0 as no pain to 10 as worst pain) pain scores were collected.

### Indication of prebiopsy prostate MRI and prostate biopsy

The indications of prebiopsy MRI to be eligible for the national reimbursement policy included elevated PSA level, presence of hypoechoic lesions on TRUS, and/or presence of a palpable nodule on digital rectal examination or PSA > 100.0 ng/ml. The need for prostate biopsy was determined via MRI findings, elevated PSA level (> 3.0 ng/ml), PSA density, presence of a palpable nodule on digital rectal examination, and/or continuous increase in PSA level during follow-up.

### MRI protocol and analysis

MRI was performed using a 3.0 Tesla system (Intera Achieva 3.0 T, Phillips Medical System, Best, Netherlands) equipped with a phased array coil (six channels). The MRI protocol involved diffusion-weighted imaging and T2 weighted imaging. T2-weighted turbo spin-echo MRI was acquired in three planes (axial, sagittal, and coronal). MRI datasets were obtained for identical slice locations, with a slice thickness of 3 mm and no intersection gap. Two b-values (0–1400) were used, and the diffusion restriction was quantified via apparent diffusion coefficient mapping. Dynamic contrast-enhanced MRI was also performed. All prostate MRIs were evaluated by an experienced urologic-radiologist and graded according to the PI-RADS Version 2.1^24^. Patients with PI-RADS scores of 3–5 were enrolled.

### Local anesthesia methods

All patients were instructed to lie in the left lateral decubitus position during the procedure. All biopsies were performed by an experienced urologist. After povidone iodine rectal preparation, 10 cc of 2% lidocaine gel was applied intrarectally (Instillagel®, Farco-Farma GmbH, Köln, Germany). After 5 min, a transrectal probe was inserted, the prostate volume was measured, and PNB was performed with a Chiba needle (A & A M.D. Inc., Seongnam, Korea).

The site of local anesthesia (base PNB vs. base and apex PNB) was determined as follows: (1) Odd days: Patients in the base PNB group received PNB on both sides of the prostate base and 2.5 cc normal saline on both sides of the prostate apex; (2) Even days: Patients in the base and apex PNB group received PNB on both sides of the prostate base as well as the prostate apex. The prostate base injections were aimed at the major neurovascular bundle after confirming the triangular echogenic “Mount Everest sign” between the prostate base and the seminal vesicle on the parasagittal longitudinal view of TRUS^25^. The prostate apex injections were aimed at a smaller triangular echogenic area between the puborectalis muscles and the prostate apex. Each PNB was performed using 2.5 cc of 2% lidocaine^9^. Patients in all groups received base injections before apex injections.

### Concurrent prostate biopsy techniques

We routinely check urine analysis and urine culture prior to biopsy decision-making. If there is pyuria or positive urine culture, sufficient antibiotics are used before the biopsy, and biopsy is performed after the follow-up urine analysis and negative confirmation of urine culture. All of the patients in this study received third-generation cephalosporin orally as prophylactic antibiotics for 2 days after the biopsy.

All biopsies were performed using the BK 3000 ultrasound system (Analogic Corporation, Peabody, MA, USA) with a 7.5–12 MHz multiplanar probe, in the following order: CBx, FBx, and SBx. First, CBx was performed with two core biopsies per target. After performing CBx, FBx was performed using the MRI/TRUS fusion system (BioJet; GeoScan, Lakewood Ranch, FL, USA) with two core biopsies per target. Therefore, four core biopsies per target were obtained. After performing CBx and FBx, SBx was performed in the order of the right lateral base, right lateral mid, right lateral apex, right medial base, right medial mid, right medial apex, left lateral base, left lateral mid, left lateral apex, left medial base, left medial mid, and left medial apex. The VAS scores were assessed at various time points: probe insertion, injection at the prostate base, injection at the prostate apex, CBx, FBx, SBx, and 15 min after prostate biopsy. We checked the VAS scores for all injections and punctures. All biopsies were performed using guide channels, which were at 19° to the transducer axis of the side-fire probe (Analogic Corporation, Peabody, MA, USA), and an 18G, 20 cm disposable core biopsy instrument (Max-Core®, CR Bard Inc., Covington, GA, USA).

### Study endpoints

The primary endpoint was VAS score for each biopsy site and PNB method. The secondary endpoints were differences in pain intensity among the biopsy methods and between the anterior and posterior sites according to CBx and FBx.

### Statistical analysis

The VAS scores for injection at the base and apex were defined as the average VAS scores for the right and left sides in base as well as apex injections. The VAS scores during CBx, FBx, and SBx were defined as the average VAS scores during individual core biopsies for the three types of biopsies.

Continuous variables are expressed as the mean ± standard deviation or median (interquartile range). Categorical variables are reported as number and frequency. The base PNB and base and apex PNB groups were compared using the independent t-test for continuous variables and the Chi-square test (Fisher’s exact test) for two or more variables. The results are presented using a linear mixed model and mean profile graph. The correlation matrix structure of the linear mixed model that showed the relationship between the collected data at various time points was calculated by applying compound symmetry. Statistical analyses were performed using SAS (version 9.4; SAS Institute, Cary, NC, USA). Statistical significance was set at *p* < 0.05.

## Data Availability

The datasets used and analyzed during the current study are available from the corresponding author on reasonable request.
